# Alternative exon usage creates novel transcript variants of tumor suppressor SHREW-1 gene with differential tissue expression profile

**DOI:** 10.1242/bio.019463

**Published:** 2016-11-15

**Authors:** Petra A. B. Klemmt, Eduard Resch, Isabell Smyrek, Knut Engels, Ernst H. K. Stelzer, Anna Starzinski-Powitz

**Affiliations:** 1 Institute of Cell Biology and Neuroscience, Department of Molecular Cell Biology and Human Genetics, Goethe Universität Frankfurt am Main, Max-von-Laue-Straße 13, Frankfurt am Main D-60438, Germany; 2 Physical Biology Group, Buchmann Institute for Molecular Life Sciences (BMLS), Goethe Universität Frankfurt am Main, Max-von-Laue-Straße 15, Frankfurt am Main D-60438, Germany; 3 Center for Pathology, Cytology and Molecular Pathology, Neuss D-41462, Germany

**Keywords:** AJAP1, Mammary gland, Lactation, Splice variants, Adherence junctions, Tumor suppressor protein

## Abstract

Shrew-1, also called AJAP1, is a transmembrane protein associated with E-cadherin-mediated adherence junctions and a putative tumor suppressor. Apart from its interaction with β-catenin and involvement in E-cadherin internalization, little structure or function information exists. Here we explored shrew-1 expression during postnatal differentiation of mammary gland as a model system. Immunohistological analyses with antibodies against either the extracellular or the cytoplasmic domains of shrew-1 consistently revealed the expression of full-length shrew-1 in myoepithelial cells, but only part of it in luminal cells. While shrew-1 localization remained unaltered in myoepithelial cells, nuclear localization occurred in luminal cells during lactation. Based on these observations, we identified two unknown shrew-1 transcript variants encoding N-terminally truncated proteins. The smallest shrew-1 protein lacks the extracellular domain and is most likely the only variant present in luminal cells. RNA analyses of human tissues confirmed that the novel transcript variants of shrew-1 exist *in vivo* and exhibit a differential tissue expression profile. We conclude that our findings are essential for the understanding and interpretation of future functional and interactome analyses of shrew-1 variants.

## Abstract (precis)

**Summary:** Transcripts of the tumor suppressor gene SHREW-1 exist in various splice variants in human and mouse encoding proteins with a differential expression and intracellular localization profile.

## INTRODUCTION

Shrew-1, also known as adherence junction-associated protein-1 (AJAP1) is a vertebrate-specific transmembrane protein characterized by an unusually long N-terminal signal peptide and one hydrophobic transmembrane segment (Bharti et al., 2004; Resch et al., 2008). In polarized cells, targeting to the basolateral plasma membrane depends on targeting motifs in the cytoplasmic tail (Jakob et al., 2006). Once delivered to the plasma membrane, shrew-1 localizes basolaterally in polarized epithelial cells and is associated with components of the adherence junction complex and a direct interaction partner of β-catenin (Bharti et al., 2004). It is implicated in modulating the internalization of E-cadherin upon growth factor stimulation in breast cancer cells (Gross et al., 2009). The function of shrew-1 in epithelial tissues remains elusive, however, it is known from progressive promoter silencing or gene deletion in brain tumors that shrew-1 can act as a tumor suppressor protein (Cogdell et al., 2011; Ernst et al., 2009; McDonald et al., 2006; Milde et al., 2009). More recent evidence indicates that the *SHREW-1/AJAP1* gene is also silenced in other tumor types such as gastric (Matsusaka et al., 2011), cervical (Chen et al., 2014) and endometrial cancer (Lai et al., 2014), or hepatocellular carcinoma (Ezaka et al., 2015).

The mammary gland is a highly regenerative organ displaying mainly postnatal development under the control of coordinated signaling events (Hennighausen and Robinson, 2001). At birth, it is a rudimentary ductal tree consisting of a bilayered epithelium composed of luminal and myoepithelial cells surrounded by stromal cells and embedded in a mammary fat pad. Ductal outgrowth and branching morphogenesis is initiated under the control of pubertal ovarian hormones to fill the entire mammary fat pad (Hennighausen and Robinson, 2005). Further differentiation occurs during pregnancy when luminal cells differentiate to milk secreting alveolar cells (alveologenesis) under the influence of growth factors and hormones such as epidermal growth factor, progesterone and prolactin (Hennighausen et al., 1997). After the lactation period, and upon cessation of suckling, the mammary gland undergoes apoptotic removal of terminally differentiated cells during involution and returns to a pre-pregnancy state. Thus, mammary gland development and function is instrumental to unravel protein expression, regulation and function in general.

EST libraries from different species and organs contain shrew-1 sequences covering different parts of the annotated shrew-1 transcript variants. This raises the possibility that shrew-1 exists in different transcript variants affecting its protein structure and/or regulation. This hypothesis is systematically addressed at the RNA and protein level both *in vitro* and *in vivo*. The mammary gland model system was chosen to characterize the expression pattern of shrew-1 and its putative protein variants in the context of epithelial differentiation.

## RESULTS

### Shrew-1 is differentially expressed in human breast tissue and during mouse mammary gland outgrowth, differentiation and involution

**Fig. 1.**
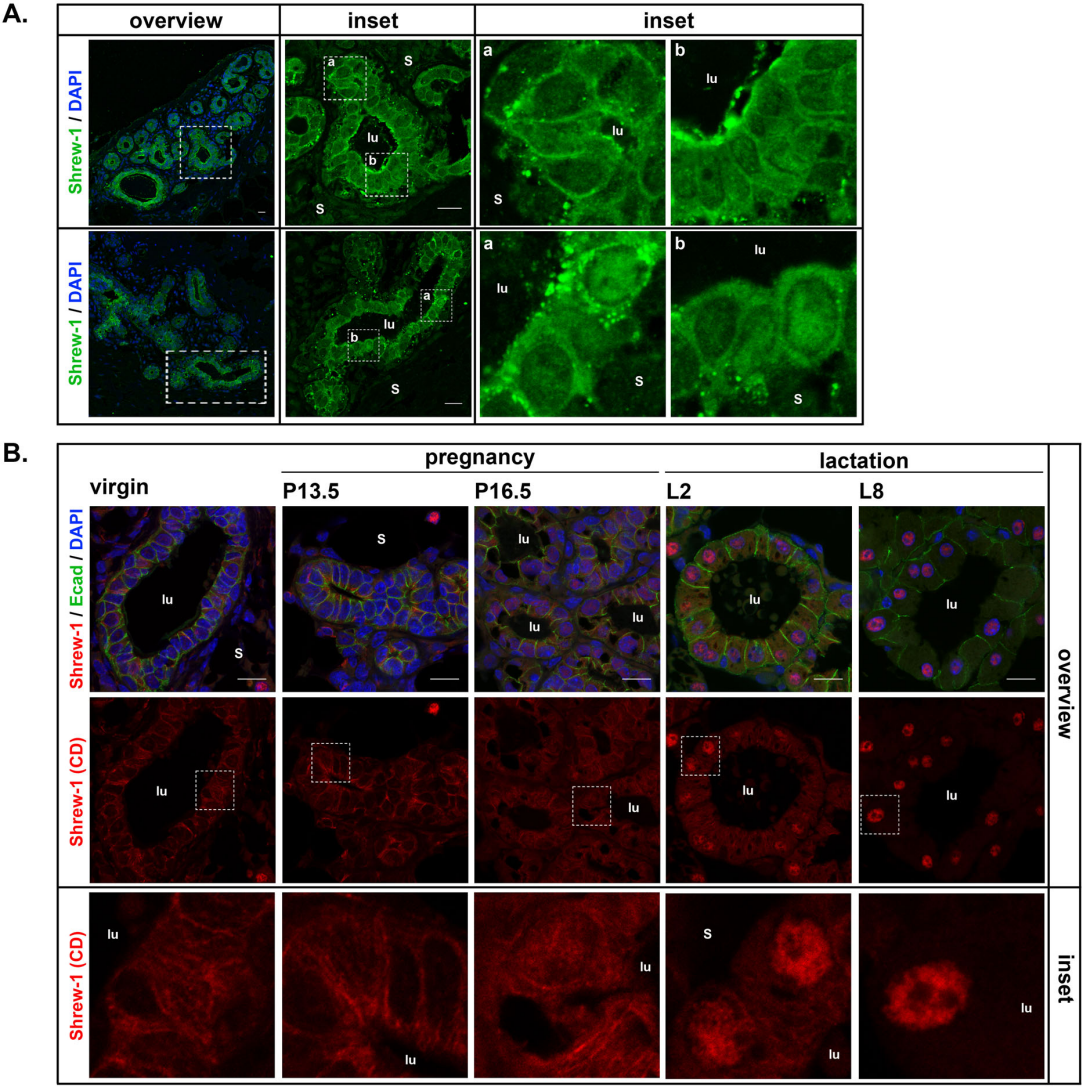
**Localization of shrew-1 in breast tissue depends on differentiation stage.** (A) Histological analysis of human breast tissue stained with a rat polyclonal anti-shrew-1 antibody (Genovac, green) revealed that shrew-1 is expressed in mammary epithelial cells in the cytoplasm and in nuclei of some luminal cells (insets). Insets represent higher magnification (zoom 4-fold). Single planes; scale bar: 15 μm; s, stroma; lu, lumen. (B) Histological analysis of mouse mammary gland tissue obtained at different developmental stages with antibodies against the cytoplasmic domain of shrew-1 (HPA012157, red) and the epithelial cell marker E-cadherin (green). Shrew-1 expression appeared cytosolic in virgin mice and displayed nuclear localization during alveologenesis and lactation. Max Z projections, scale bar: 15 μm; s, stroma; lu, lumen. Blue in A and B: DNA stained with DAPI.

We reported that shrew-1 is located at cell-cell contacts in human breast tissue (Gross et al., 2009). A different intracellular localization of shrew-1 was observed in healthy appearing mammary tissue of poorly differentiated invasive ductal carcinoma. Here shrew-1 was not located at cell-cell contacts but appeared within the cytoplasm of mammary epithelial cells (Fig. 1A). In addition, we detected shrew-1 in some nuclei of luminal epithelial cells (Fig. 1A insets). Based on these findings and the availability of several novel antibodies raised against different protein domains of shrew-1 (Table 1 and Fig. S1), we investigated its localization and distribution pattern in a well-defined model system for epithelial differentiation, i.e. the mouse mammary gland. The developmental stages of mouse mammary gland comprise of postnatal outgrowth during puberty, and repetitive pregnancy cycles marked by alveologenesis, lactogenesis and involution upon cessation of suckling (Hennighausen and Robinson, 2001; Inman et al., 2015).

**Table 1.**
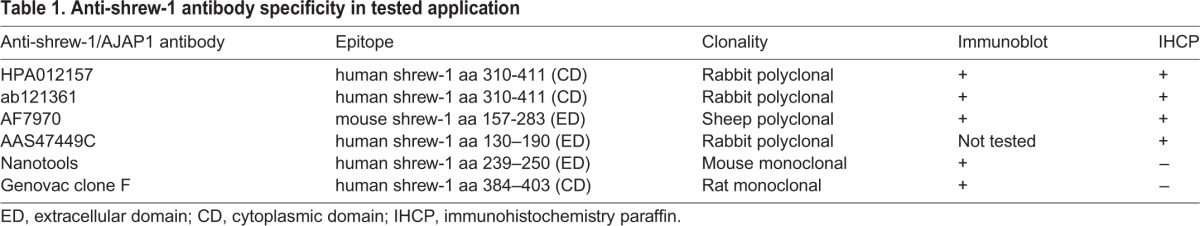
**Anti-shrew-1 antibody specificity in tested application**

We performed immunohistological analyses of mouse mammary gland tissue sections collected at different developmental stages (virgin, pregnancy and lactation) initially with a polyclonal antibody against the cytoplasmic domain of shrew-1. Comparable to human mammary gland (Fig. 1A), we detected the expression of shrew-1 in the bilayered mammary epithelium, which is positive for E-cadherin expression (Fig. 1B). However, the localization of shrew-1 in murine mammary gland varied in the myoepithelium and luminal cells between the different developmental stages. Shrew-1 localized within the cytoplasm of virgin and mid-pregnant mice in both epithelial cell layers; however, its localization changed in luminal cells of late-pregnant mice [P16.5 days post coitum (dpc)]. Here shrew-1 is detectable in the cytoplasm and in the nuclei of luminal cells, and this nuclear localization became more pronounced with the onset and duration of lactation. The nuclear appearance of shrew-1 in mouse mammary glands coincided with phosphorylation and nuclear translocation of a physiological marker for lactogenesis, signal transducer and activator of transcription 5 (STAT5, Fig. S2A).

**Fig. 2.**
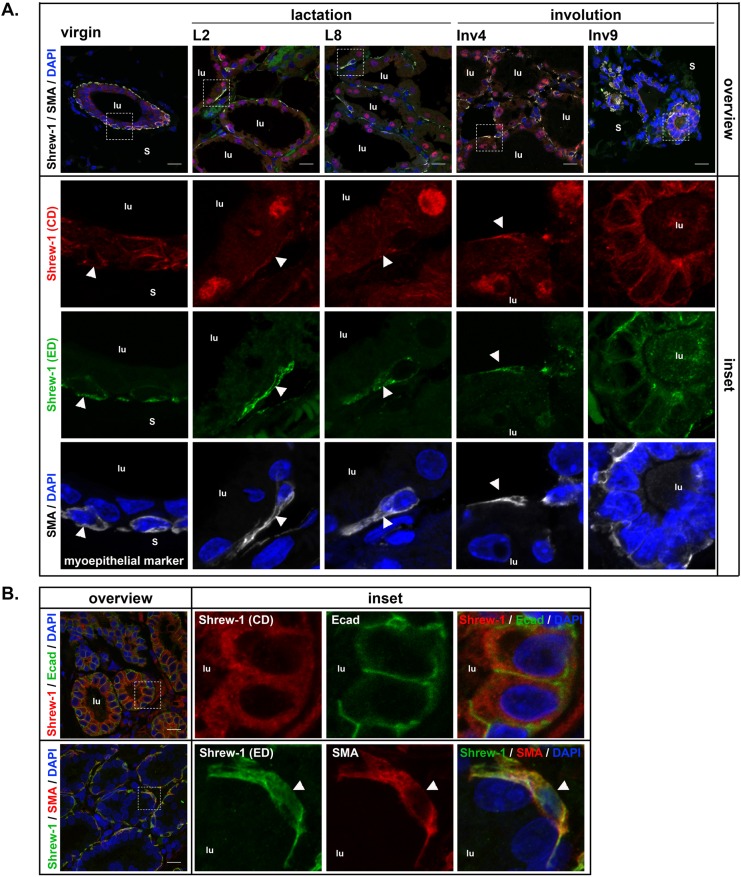
**Differential localization of shrew-1 in mammary epithelial cell layers indicates novel shrew-1 protein isoforms.** (A) Histological analyses of shrew-1 localization in murine mammary gland tissue with polyclonal anti-shrew-1 antibodies against the cytoplasmic (CD, ab121361, red) or extracellular domain (ED, AF7970, green) in combination with the marker for myoepithelial cells smooth muscle actin (SMA, gray). Colocalized staining with both anti-shrew-1 antibodies could only be observed in myoepithelial cells, whereas luminal cells were only stained with the anti-shrew-1 CD antibody. The nuclear localization of shrew-1 in luminal cells observed during alveologenesis and lactation persisted during early stages of involution. By later stages of involution (Inv9) shrew-1 was present in the cytoplasm. Insets represent higher magnification (zoom 4.8-fold). Max Z projections; S, stroma; lu, lumen; white arrowhead, myoepithelial cell; scale bar: 15 μm. (B) Histological analyses of human breast adenoma tissue with antibodies against the cytoplasmic domain of shrew-1 (CD, ab121361) in combination with E-cadherin (green) or the extracellular domain of shrew-1 (ED, AF790) in combination with SMA (red). Shrew-1 localization in luminal cells is only detected with the anti-shrew-1 CD antibody whereas colocalized staining with both anti-shrew-1 antibodies is observed in myoepithelial cells. Max Z projections; lu, lumen; white arrowhead, myoepithelial cell; scale bar: 15 μm. Blue in A and B, DNA stained with DAPI.

In a second immunohistological analyses we used polyclonal antibodies either against the extracellular (ED) or the cytoplasmic domain (CD) of shrew-1 in combination with the myoepithelial marker smooth muscle actin (SMA, Fig. 2A). We detected co-immunostaining with both antibodies in the myoepithelium in all mammary gland differentiation stages suggesting the presence of the full-length shrew-1 protein. Unexpectedly, immunostaining for shrew-1 in luminal cells was present only with the antibody against the CD. The lactation-dependent emerging nuclear localization of shrew-1 in luminal cells persisted during early involution (day 1 and 4) (Fig. 2A and Fig. S2B) alongside the nuclear localization of a physiological marker for involution, i.e. phosphorylated STAT3 (Fig. S2B,C). During the later stages of involution (day 9), the nuclear staining of STAT3 was lost (Fig. S2D) and shrew-1 staining in luminal cells was again cytoplasmic (Fig. 2A), comparable to its localization observed in virgin mice.

In order to assess whether the differential antibody recognition also occurs in humans, we performed immunohistochemical analysis of healthy human breast tissue. Similar to the mouse mammary gland, co-immunostaining of shrew-1 was only seen in myoepithelial cells, but not in luminal cells (Fig. 2B). The differential staining pattern of shrew-1 supports the hypothesis that a modified shrew-1 protein species exists in luminal mammary epithelium, which either lacks the extracellular part or underlies post-translational modification, thus masking the epitope binding sites in luminal cells.

The specificity of the available shrew-1 antibodies was tested with relevant methods, for example selective detection of shrew-1 protein isoforms in immunoblot and for immunohistology with antibody preabsorption prior to commencing with the staining procedure (Fig. S1).

### Identification of novel shrew-1 transcript variants and their expression pattern in different organs

**Fig. 3.**
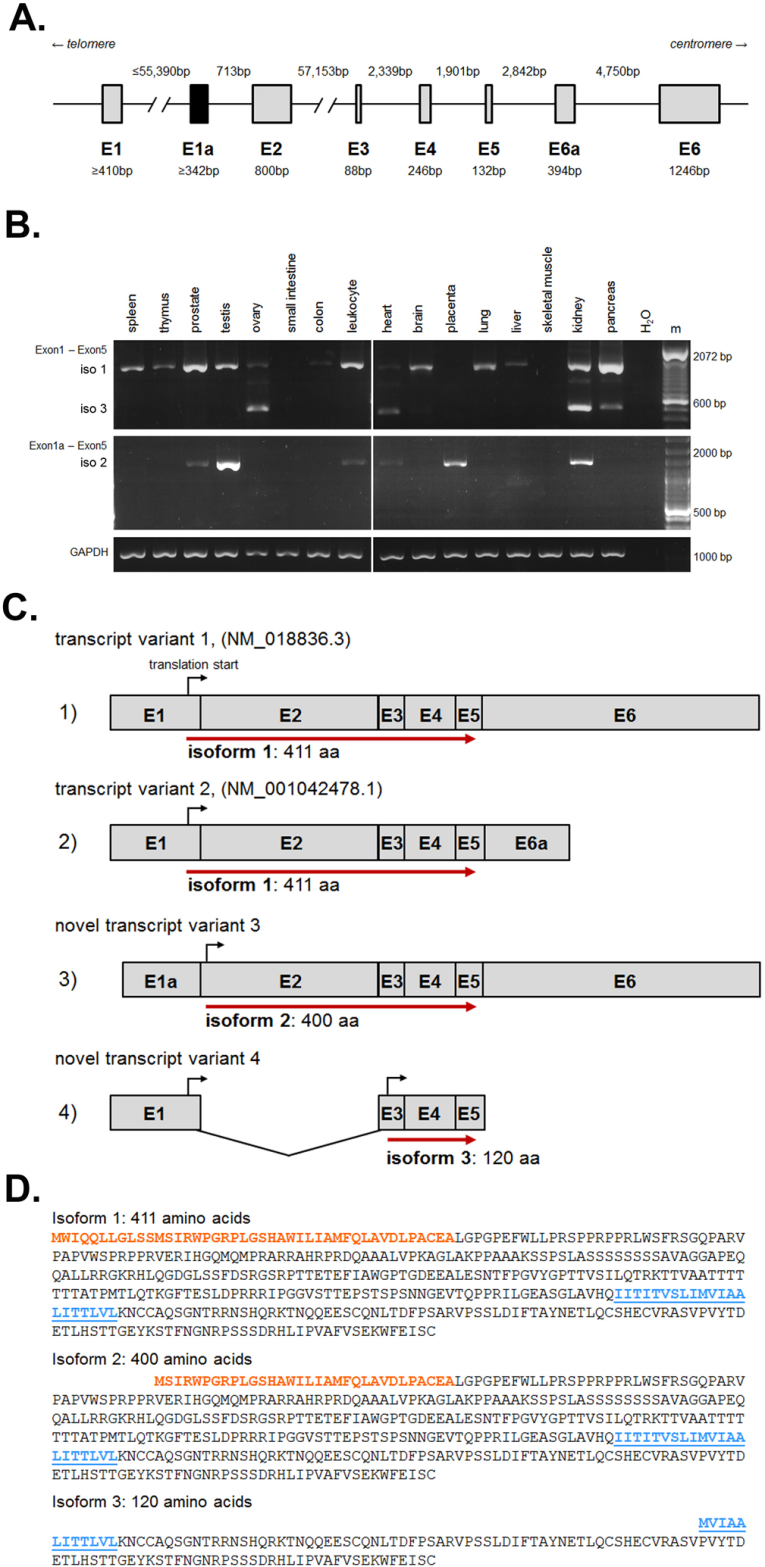
**Alternative exon usage creates shrew-1 transcript variants coding for three different protein isoforms with organ specific expression patterns.** (A) The human *SHREW1/AJAP1* gene, encompassing about 129,000 bp, is encoded on chromosome 1p36.32 in seven annotated exons (E1 to E6, gray boxes) and a novel one (E1a, black box). The lengths of the introns are given above and exons below. The schematic drawing is based on the alignment of the human shrew-1 genomic sequence (NC_000001.10, position 4715104-4843850) with the two shrew-1 transcripts (NM_018836.3, NM_001042478.1) in line with the novel E1a. (B) cDNAs of several human organs were used to analyze the shrew-1 transcript variant expression. Using primers that bind to exons E1 and E5, the transcript variant encoding shrew-1 isoform 1 was detectable by PCR (1308 bp). The band emerging at 508 bp is a novel transcript variant lacking E2. The transcript encoding shrew-1 isoform 2 (1299 bp) was detected with an E1a-specific forward primer together with the reverse primer binding to E5. GAPDH, the transcript of a housekeeping gene was amplified as a positive control; m, DNA ladders. (C) (1) NM_018836.3 and (2) NM_001042478.1 are the two known shrew-1 transcript variants, which differ in their 3′-untranslated region by alternative exon usage (E6 and E6a, respectively). The open reading frame (ORF) of the encoded shrew-1 protein (E1 to E5, red arrow) is not affected and gives rise to a 411 aa residue long peptide (isoform 1). (3) Alternative usage of E1a instead of E1 leads to an N-terminal truncation of the first 11 aa residues (isoform 2). (4) Alternative splicing of E1 to E3 thus skipping E2, results in an N-terminally truncated protein isoform lacking the entire ED and part of the TMS (isoform 3). (D) Primary structure of the protein isoforms 1-3. Isoform 1 is the published shrew-1 protein encompassing 411 aa residues. Isoform 2 is N-terminally truncated resulting in a shortened SP (orange) and a protein of 400 aa residues in length. Isoform 3 is a putative 120 aa residues long protein starting in the TMS (blue, underlined) of isoform 1 and 2, respectively.

Shrew-1 expression has been confirmed in several organs, e.g. pancreas, uterus, brain and mammary gland (Bharti et al., 2004; Gross et al., 2009; McDonald et al., 2006). To date, two human shrew-1 mRNA transcripts (NM_018836.3, NM_001042478.1) are annotated in the NCBI RefSeq database (Pruitt et al., 2012) based on the entries in the GenBank (www.ncbi.nlm.nih.gov/genbank/). These two transcripts are composed of seven shrew-1 exons (E1, E2, E3, E4, E5, E6, and E6a) (Fig. 3A, gray boxes). As the open reading frame (ORF) of shrew-1 reaches from E1 to E5, the alternative usage of E6 and E6a does not affect the protein coding sequence. Thus, transcript variants 1 and 2 encode the 411 amino acid (aa) residues long shrew-1 protein, which we refer to as shrew-1 protein isoform 1. We identified a further exon of shrew-1, namely E1a, which is located in the *SHREW-1/AJAP1* gene between E1 and E2 (Fig. 3A, black box; Fig. S3A). Alternative splicing of this E1a to E2 (instead of E1) results in a novel transcript, transcript variant 3. E1a lacks a translation initiation codon so that the next possible translation initiation codon is located on E2. The protein synthesized from this translation initiation codon lacks the first 11 aa residues of shrew-1 protein, whereas the remaining 400 aa residues are identical to it (shrew-1 protein isoform 2).

We used a selection of human cDNA libraries generated from 16 different organs in order to analyze the mRNA expression profile of the identified shrew-1 transcript variants so far (Fig. 3B). Two primer pairs were designed to amplify specifically either the cDNA encoding shrew-1 protein isoform 1 (E1 to E5; 1308 bp) or the cDNA encoding shrew-1 protein isoform 2 (E1a to E5; 1299 bp). Expression of shrew-1 encoding isoform 1 was detectable in most analyzed organs (Fig. 3B). In addition, for the first time, the expression of isoform 2-encoding shrew-1 transcript was verified *in vivo*, encompassing the complete protein coding sequence (Fig. 3B). This transcript was detectable in prostate, testis, leukocyte, heart, placenta and kidney (verified by sequencing of obtained RT-PCR products). Placenta was the only sample exclusively expressing the transcript of isoform 2. In several organs (ovary, heart, kidney, pancreas and brain), an unexpected PCR product of only 508 bp was noted with the primer pair to detect shrew-1 transcripts of isoform 1. Subsequent sequencing of this PCR product revealed a novel alternative transcript splice variant of shrew-1 precisely lacking the sequence of the 800 nucleotide (nt) long E2 (Fig. 3C and Fig. S3B). This finding suggests that a so far unknown additional shrew-1 mRNA transcript exists, which is a truncated variant of the shrew-1 protein isoforms 1 and 2 covering the rear stretch of the transmembrane segment (TMS) and the following CD (isoform 3; Fig. 3C; transcript variant 4).

Based on these data, we refer to the long version of shrew-1 (Fig. 3D) as shrew-1 isoform 1 (411 aa, encoded by transcript variants 1 and 2), the N-terminally truncated shrew-1 protein as isoform 2 (400 aa, transcript variant 3) and the even shorter transcript variant 4 as isoform 3 with 120 aa.

### Shrew-1 isoform 2 retains plasma membrane delivery and cell surface presentation, whereas isoform 3 is an intracellular membrane-associated protein

**Fig. 4.**
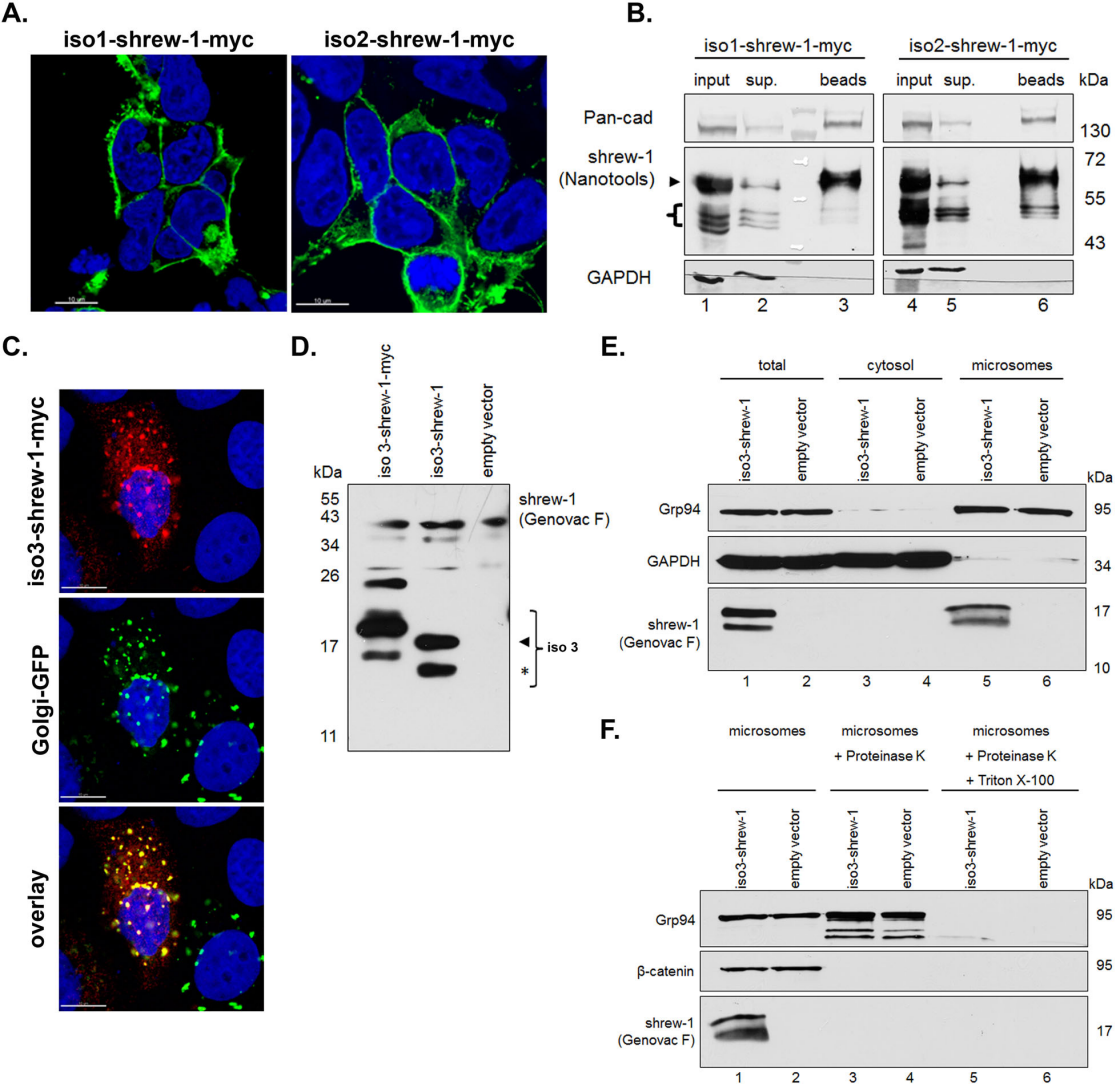
**Shrew-1 protein isoforms 2 and 3 are targeted to the secretory pathway.** (A) Constructs encoding iso1-shrew-1-myc and iso2-shrew-1-myc were expressed in HEK293T cells and detected with an anti-shrew-1 antibody (Nanotools, green). Nuclei stained with DAPI (blue). Both shrew-1 protein isoforms predominantly localized at the plasma membrane. Scale bar: 10 μm. (B) HEK293T cells expressing iso1-shrew-1-myc or iso2-shrew-1-myc were probed with biotin, pulled down with NeutrAvidin beads and analyzed by SDS-PAGE and immunoblot (beads) alongside 13% each of the pull-down input (input) and supernatant (sup) and visualized with an anti-shrew-1 antibody (Nanotools). Comparable pull-down efficiencies and purity of the cell surface samples was confirmed with Pan-cadherin and GAPDH detection. Both shrew-1 protein isoforms are O-glycosylated (arrowhead; Fig. S4 and main text), but also exhibit a fraction of premature protein (bracket). (C) Co-transfection of MCF-7 cells with iso3-shrew-1-myc (Genovac F, red) and Golgi-GFP showed a co-localization of iso3-shrew-1-myc with the trans cisternae of the Golgi apparatus (yellow). The nuclei were stained with DAPI (blue). Scale bar: 10 μm. (B) Ectopic Shrew-1 isoform 3 expression in HEK293T cells revealed the presence of double bands at the estimated size of 13.4 kDa (asterisk) and at about 17 kDa (arrowhead) compared to the empty vector control (bracket). Shrew-1 isoform 3 fused with a myc-tag (1.2 kDa) exhibited for both bands a delayed migration. (D) Cytosol-microsomal fractionation of transfected HEK293T cells was performed followed by immunoblot analysis of both fractions (cytosol, microsomes) as well as whole cell lysate samples (total). As markers for the distinct fractions GAPDH (cytosol) and Grp94 (microsomes) were visualized. Shrew-1 isoform 3 was detected in the microsomal fraction as a double band. (E) The isolated microsomal membranes of iso3-shrew-1 or empty vector transfected HEK293T cells were subjected to a proteinase K-protection assay. Whereas Grp94, the ER-lumenal protein, was protected by microsomal membranes from the proteolytic digestion, iso3-shrew-1 and β-catenin, associated with the membrane at the cytosolic side, were not. (F) By addition of Triton X-100 the microsomal membranes were disrupted, resulting in the degradation of Grp94. These results suggest that shrew-1 isoform 3 is associated with the membrane at the cytosolic side.

It was shown previously that the targeting of shrew-1 isoform 1 to the cell surface depends on its signal peptide (SP) and the TMS (Resch et al., 2008). The discovery of the novel exon 1a, and the natural occurrence of the N-terminally truncated shrew-1 protein isoform 2 with a shorter signal peptide, raised the question whether this protein isoform is delivered to the cell surface as efficiently as isoform 1. Immunofluorescence staining of HEK293T cells transfected with either iso1-shrew-1-myc or iso2-shrew-1-myc showed prominent plasma membrane localization with a similar distribution pattern (Fig. 4A), which was confirmed by cell surface biotinylation for both isoforms (Fig. 4B). In contrast, the novel shrew-1 isoform 3 lacking the SP, the ED, and parts of the TMS is considerably smaller in size (13.4 kDa) than the other shrew-1 isoforms (precursor size approx. 44.5 kDa). Moreover, as isoform 3 lacks the SP and the truncated TMS is shunted to its N-terminus (Fig. 3D), we next addressed where this shrew-1 protein isoform would localize within the cell. *In silico* analysis using *SignalP* (Petersen et al., 2011) predicted a SP of 17 aa residues in length for isoform 3 with a SP cleavage site in the former CD of shrew-1 isoforms 1 and 2 (data not shown). Thus, the truncated TMS, together with the following five aa residues, were thought to target and translocate the former CD across the ER membrane as the TMS is able to target shrew-1 to the secretory pathway on its own (Jakob et al., 2006). In order to test this prediction experimentally, the cDNA of shrew-1 isoform 3 was cloned into an expression vector and transfected into MCF-7 cells. Immunofluorescence staining using the anti-shrew-1 antibody (Genovac F) raised against the CD of shrew-1 isoform 1 revealed that shrew-1 isoform 3 was distributed in the cytoplasm, thereby accumulating in dotted structures (Fig. 4C). Co-expressed GFP fused to the N-terminal signal-anchor of the human β-1,4-galactosyl-transferase was used as a marker for the trans cisternae of the Golgi apparatus showing that shrew-1 isoform 3 colocalized with these structures. Immunoblot analysis of exogenously expressed shrew-1 isoform 3 in HEK293T cells revealed that two distinct protein bands were detectable for the protein (Fig. 4D). The lower migrating band emerged at about the expected size of 13.4 kDa, or somewhat higher (asterisk), whereas the higher migrating band emerged at a considerably larger size than expected, about 17 kDa (arrowhead). Analogously, the same double band was observed for shrew-1 isoform 3 fused to a myc-tag at the C-terminus. As expected, iso3-shrew-1-myc exhibited a shift caused by the 1.2 kDa tag, thus supporting the estimated size of the upper and the lower bands. Next we assessed whether exogenously expressed iso3-shrew-1 in HEK293T cells could be found associated with the secretory pathway and performed cell fractionation experiments (Fig. 4E). In immunoblot analysis of cytosolic and microsomal fractions using the anti-shrew-1 antibody (Genovac F), iso3-shrew-1 emerged as two distinct protein bands exclusively in the microsomal fraction comparable to non-fractionated cells (Fig. 4E). To validate the luminal localization of iso3-shrew-1, the isolated microsomes were subjected to a proteinase K protection assay (Fig. 4F). Unexpectedly, the proteinase K protection assay revealed that, in contrast to the *in silico* prediction, iso3-shrew-1 was not luminal, but was associated with the membrane from the cytosolic side as iso3-shrew-1 was degraded alongside β-catenin but not Grp94, which served as control. However, the addition of Triton X-100 in combination with proteinase K degraded Grp94, indicating that the microsomes were intact and protected luminal proteins under proteinase K digestion. These data suggest that shrew-1 isoform 3 is not a secreted protein as predicted, but instead is targeted to the membranes of the secretory pathway and remains attached to them. These findings imply that shrew-1 isoform 3 retains the same orientation with regard to the membrane as the CD of shrew-1 isoforms 1 and 2.

### Detection of Shrew-1 isoforms 1 and 3 in several murine organs

**Fig. 5.**
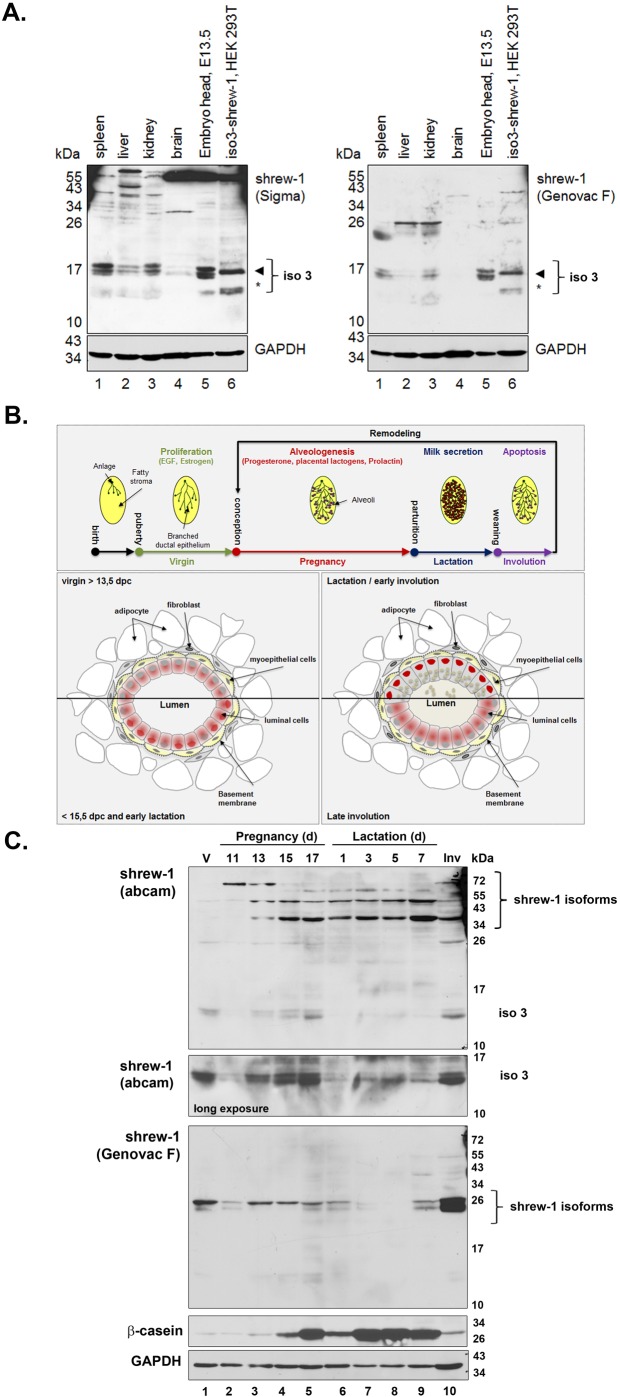
**Detection of shrew-1 isoforms in mouse organs.** (A) Protein lysates of several murine organs were subjected to SDS-PAGE and analyzed by immunoblot using antibodies recognizing the CD of shrew-1, Sigma and Genovac F, respectively. Shrew-1 isoform 3 exogenously expressed in HEK293T was used as positive control and GAPDH as loading control. Both the Genovac F antibody as well as the Sigma antibody recognized the double bands of iso3-shrew-1 (13.4 kDa, asterisks; 17 kDa, arrowhead). Interestingly, also in the organs (spleen, kidney, embryo head, and to a lesser amount in the liver and the brain) protein species at about 17 kDa (arrowhead) were detected by both antibodies, suggesting the existence of a shrew-1 protein with the size of isoform 3 *in vivo*. (B) Schematic overview of mammary gland differentiation cycle and intracellular localization of shrew-1 isoforms by histological analysis. (C) Whole fat pad lysates of murine mammary gland at different developmental stages were subjected to SDS-PAGE and analyzed by immunoblot using antibodies recognizing the CD of shrew-1 to visualize all known shrew-1 isoforms (abcam and Genovac F). The antibody against β-casein was used as differentiation marker and probing against GAPDH as loading control. A double protein band at approx.13 kDa representing isoform 3 could be detected with the shrew-1 abcam antibody (long exposure) and additional bands ranging from 34 to 72 kDa possibly representing isoform 1 expression. The Genovac F antibody recognized a double band at approx. 26 kDa. d, days; V, virgin; Inv, involution.

Interestingly, an expressed sequence tag (EST) clone exactly matching the novel transcript variant encoding shrew-1 protein isoform 3 exists for *Mus musculus* (BI990953.1). This indicates that the observed differences in antibody recognition between myoepithelial and luminal cells might be explained by the presence of different shrew-1 protein variants in murine and human mammary glands (Fig. 2). To address the question of whether shrew-1 protein isoform 3 could be detected *in vivo*, several murine organs were lysed and subjected to immunoblot analysis with two different antibodies against the CD recognizing different epitopes (Table 1 and Fig. S1) of shrew-1 on separate membranes (Fig. 5A). HEK293T cells exogenously expressing shrew-1 isoform 3 served as a positive control. Interestingly, both antibodies recognized proteins of the size of isoform 3 in the organs, with the strongest signal found in the spleen, the kidney, and the embryo head (Fig. 5A). In contrast to the exogenously expressed isoform 3, the proteins appearing in the organ samples displayed a double or triple band. As they exhibited the size of the upper isoform 3 band of about 17 kDa, it suggests that they might also be post-translationally modified. The data indicate that a shrew-1 protein with the size, and presumably with the modifications, of isoform 3 exists *in vivo*. In order to align the immunostaining data with the putative shrew-1 protein expression during mammary gland differentiation and remodeling (Fig. 5B), we analyzed the presence of shrew-1 transcript variants in whole mammary gland protein extracts (Fig. 5C). Interestingly, we observed several protein bands ranging from approximately 13 kDa to 72 kDa with the polyclonal antibody against the CD of shrew-1 (abcam). Longer exposure times indicated that protein bands for isoform 3 were present. The higher ranging bands correlated with the expression of shrew-1 isoform 1. In contrast, only a signal at approximately 26 kDa was obtained with the monoclonal antibody Genovac F raised against the CD of shrew-1. The expected molecular weight of shrew-1 protein isoforms 1 and 3 are 44.5 kDa and 13.4 kDa, respectively. The obtained band pattern suggests expression of both shrew-1 protein variants that might have undergone post-translational modification which might affect the recognition by the two antibodies.

### Predominant expression of shrew-1 protein isoform 3 is in mammary epithelium

**Fig. 6.**
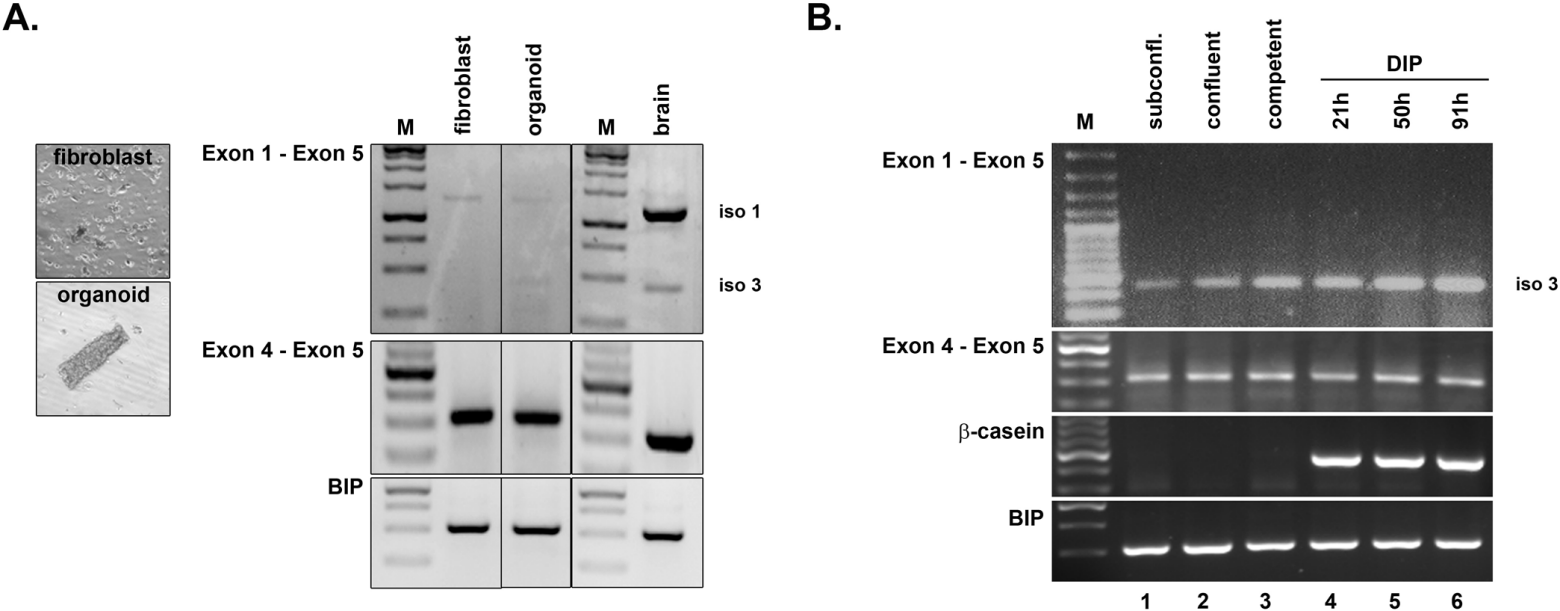
**Detection of shrew-1 transcript variant expression during murine mammary gland differentiation.** (A) Tissue digest of whole fat pads from a virgin mouse was performed to separate mammary organoids and fibroblasts. RT-PCR was performed with a primer pair to detect protein isoforms 1 and 3 (E1 to E5, isoform 1: 1269 bp; isoform 3: 463 bp). The presence of shrew-1 cDNA was confirmed with a primer pair to amplify E4, which is common to all known transcript variants (E4 to E5; 323 bp). cDNA of brain tissue (E13.5) served as positive control for both shrew-1 transcript variants encoding shrew-1 protein isoforms 1 and 3 respectively; the housekeeping gene BIP served as positive control for cDNA synthesis. cDNA analysis showed that both shrew-1 transcript variants corresponding to protein isoforms 1 and 3 are present in organoids but only isoform 1 is detectable in fibroblasts. M, bp marker. (B) The lactogenic inducible mammary epithelial cell line HC11 was differentiated *in vitro* with a hormone cocktail (DIP). RT-PCR with primer pairs to detect shrew-1 transcript variant expression (E1 to E5, isoform 1: 1269 bp hash; isoform 3: 463 bp delta) or the presence of all known shrew-1 transcript variants (E4 to E5; 323 bp) indicate that only the shrew-1 transcript encoding protein isoform 3 is expressed during all differentiation stages (delta). M, bp marker.

The whole fat pad protein lysates of mammary tissue collected at different developmental stages confirmed the presence of shrew-1 protein but revealed a discrepancy between deduced protein size of the identified transcript variants. As mammary fat pads consist of a bilayered mammary epithelial tree surrounded by fibroblasts and adipocytes, we digested mammary gland tissue to analyze shrew-1 transcript expression in purified mammary organoids (consisting of myoepithelial and luminal cells) and fibroblasts (Fig. 6A). RT-PCR was performed with a primer pair to detect specifically the cDNA transcript variants for protein isoform 1 (1269 bp) and 3 (463 bp) expression in one reaction (primer pair from E1 to E5). As a control for shrew-1 cDNA expression in general, exon 4 was amplified with a primer pair that is common to all identified shrew-1 transcript variants so far in *Mus musculus* (primer pair from E4 to E5; 323 bp). Brain tissue served as a positive control for shrew-1 expression, and the housekeeping gene BIP as a positive control for cDNA synthesis. Both shrew-1 transcript variants encoding protein isoform 1 and 3 respectively could be detected in mammary organoids, whilst in fibroblasts only the long shrew-1 transcript variant encoding protein isoform 1 could be detected. Next, we addressed the question of whether luminal cells indeed only express the novel shrew-1 protein isoform 3 as suggested by the obtained histological analysis (Figs 1B and 2A). For this, we used HC11 cells, a murine mammary luminal epithelial cell line capable of lactogenic differentiation (Ball et al., 1988). HC11 cells were differentiated with a hormone cocktail containing dexamethasone, insulin and prolactin (DIP) for several days to induce alveologenesis as previously described (Vafaizadeh et al., 2010). The mRNA expression at different stages of lactogenic differentiation confirmed that only the transcript encoding the shrew-1 protein isoform 3 is present in luminal cells (Fig. 6B).

### Post-translational modification of shrew-1 affects protein size

In previous reports (Bharti et al., 2004; Gross et al., 2009; Jakob et al., 2006; Schreiner et al., 2007), as well as in this study, shrew-1 protein emerged in immunoblot analysis about 15 to 20 kDa above the expected size, raising the question about the nature of this apparently increased molecular weight. This could be due to several possibilities such as transcript variant expression or post-translational modifications. Glycosylation of the protein was suggested, as it is known that glycans reduce the SDS binding of proteins and subsequently the reduced charge-to-mass ratio slows down the migration of the protein in the SDS-PAGE (Poduslo, 1981).

Using the prediction software NetOGlyc v3.1 (Julenius et al., 2005), 34 putative O-glycosylation sites were identified in the ED of shrew-1 (Fig. S4A). Human shrew-1 protein exhibits three clusters in the ED with serines and threonines predicted to be O-glycosylated, encompassing the peptide stretches from the positions 126 to 137, 211 to 237, and 251 to 266 in the shrew-1 sequence (Fig. S4A). Moreover, the clusters are largely conserved in shrew-1 orthologues and were also predicted to be O-glycosylated (data not shown). To test the possible O-glycosylation of shrew-1, an O-glycosylation mutant was generated substituting putative O-glycosylation sites (threonines at the positions 230, 233 and 237 as well as serines at the positions 239 and 251) by alanines. The O-glycosylation mutant, as well as wild-type shrew-1 (isoform 1), were expressed in MDCK cells. The immunoblot analysis of both shrew-1 proteins showed that the substitution of these five aa residues affected the apparent size of the shrew-1 protein (Fig. S4B). The shrew-1 O-glycosylation mutant appeared with a distinct smaller size (delta) compared to the wild-type protein (arrowhead). Apparently, by targeting these amino acid residues, relevant O-glycosylation sites were hit, resulting in a reduced O-glycosylation and, therefore, in an apparent size closer to the theoretical size (44.5 kDa) of the premature precursor protein.

Next, we treated lysates of MDCK cells stably expressing shrew-1 (isoform 1) with acetylneuraminyl hydrolase (neuraminidase) alone, or additionally with endo-α-N-acetylgalactosaminidase (O-glycosidase). A slight downshift of shrew-1 protein was observed after neuraminidase treatment (Fig. S4C, lane 2) and subsequent additional digestion with O-glycosidase resulted in a dramatic shift of the shrew-1 protein down to the calculated size of the precursor (brace; Fig. S4C, lane 3).

## DISCUSSION

We describe the discovery of two so far unknown transcript variants of the tumor suppressor shrew-1. Both novel transcripts arise by alternative exon usage. The originally identified shrew-1 transcript encodes a protein of 411 aa (isoform 1) with an unusually long signal peptide (SP, aa 1-43) that contains functional distinct targeting subdomains (Bharti et al., 2004; Hiss et al., 2008). The first newly identified shrew-1 transcript variant (referred to as isoform 2) starts with a novel exon (E1a), leading to an 11 aa shorter SP, that might bring so far unknown subcellular targeting features to shrew-1. Notably, the NCBI RefSeq database (Pruitt et al., 2012) predicts a shrew-1 protein isoform X2 (XP_011540089), which is identical to our newly discovered isoform 2. However, the putative transcript encoding isoform X2 (XM_011541787) contains additional information next to our identified E1a sequence.

The second novel shrew-1 transcript (referred to as shrew-1 isoform 3) contains E1 (such as the transcript for isoform 1), but lacks E2. This transcript encodes an even shorter N-terminally truncated shrew-1 protein, which lacks the entire extracellular domain (SP and ED) and part of the transmembrane segment (TMS). The data generated on isoform 3 imply its natural occurrence *in vivo* and might have shared or different functions to the other isoforms.

For a better understanding of the primary structure-function relation of shrew-1's isoform 3, we performed *in silico* analyses (*SignalP*, *Splign*). These predicted a functional SP, and a SP cleavage site between the aa residues 17 and 18. Experiments analyzing exogenously expressed shrew-1 isoform 3 suggest that it targets to the secretory pathway. However, it apparently attaches from the cytosolic side to the membrane rather than translocating across the membrane. The remaining TMS of shrew-1 isoform 3 encompasses 12 aa residues, posing the question of whether this stretch may be sufficient and responsible for the anchorage of the protein to the membrane. Typically, TMSs are about 21 aa residues in length (Senes et al., 2000), but there are also very short segments known, for example found in aquaporins (Sui et al., 2001) or H+/Cl- - exchangers (Dutzler et al., 2002). Based on these and other studies available (Dutzler et al., 2002; Jaud et al., 2009; Krishnakumar and London, 2007; Senes et al., 2000; Sui et al., 2001), it is conceivable that shrew-1 isoform 3 is anchored in the membrane via its truncated TMS.

An increasing number of genetic studies of tumors of the brain (glioblastomas, neuroblastomas, ependymom), and also of non-brain cancers (cervical, endometrial, squamous cancers), imply that shrew-1 protein is of functional relevance in physiology and pathophysiology. According to these analyses, shrew-1 (also called AJAP1) is a tumor suppressor whose gene becomes hypermethylated and thus epigenetically silenced in the course of tumor development (Chen et al., 2014; Cogdell et al., 2011; Ezaka et al., 2015; Lai et al., 2014). In some cases described for glioblastoma, loss of function of shrew-1 occurs through deletions of the respective genomic region chr1p36 harboring its gene (Ernst et al., 2009; Lin et al., 2012; McDonald et al., 2006; Milde et al., 2009). Altogether, these findings support the idea that the *SHREW-1/AJAP1* gene encodes functionally important protein(s) involved in regulatory protein circuits whose alterations contribute to tumor development and to so far unknown physiological processes.

A common feature of all so far discovered shrew-1 variants is the presence of the complete or partial TMS and the entire cytoplasmic tail. It could, for example, harbor interaction domains for the formation or the cross-linking of signaling platforms, and several observations are in line with such a statement. Previously, we described that shrew-1 is present in protein complexes together with proto-oncogene SRC and epidermal growth factor (EGF) receptor tyrosine kinase HER-2 in MCF-7 breast cancer cells after growth factor stimulation with EGF. In these types of experiments, shrew-1 obviously modulates the surface levels of tumor suppressor E-cadherin (Gross et al., 2009).

In addition, we have shown that shrew-1 can target to E-cadherin-adherence junctions (AJ) of polarized MDCK cells in a tightly regulated manner. This activity involves interaction (direct or indirect) with μ1B, a subunit of the epithelial specific adaptor complex AP-1B and specific aa in shrew-1's cytoplasmic domain (CD). Another prominent protein of AJs that can interact directly with shrew-1 is β-catenin, a major signaling protein of the WNT signaling pathway (Bharti et al., 2004). In principle, shrew-1 and β-catenin can be constituents of AJs and the nucleus; therefore, it might be possible that they interact in either structure or organelle, respectively. Notably, so far we only have experimental evidence that shrew-1's isoform 3 targets to the nucleus (this study), however, this does not exclude that another isoform or part of it also targets the nucleus.

Currently, hardly any knowledge exists in which functional context the ED of shrew-1 is of importance. Our previous experiments have indicated that shrew-1's ED is obviously dispensable for its basolateral targeting since shrew-1 without its ED integrates into AJs, although gives rise to less defined localization (Jakob et al., 2006). The first insight into a putative function of the shrew-1 ED as a binding partner for receptor complex composition comes from a recent publication on mammalian brain extracts. Here, proteomic data indicate that shrew-1 peptides from both the extracellular and cytoplasmic domain could interact with GABA_B_ receptor subunits (Schwenk et al., 2015). Since we show that shrew-1's ED is a target for O-glycosylation, it would be of high interest to investigate to what extent changes in the composition of the attached glycans may affect shrew-1's ED protein-protein interaction.

The mouse mammary gland is an informative model system allowing analysis of shrew-1 isoforms *in vivo* at different developmental stages. The fact that shrew-1 detection by antibodies differed within the epithelium and during functional differentiation indicated that at least two protein isoforms of shrew-1 are present. A colocalization signal of the antibody staining for shrew-1 against the ED and CD in myoepithelial cells suggests that shrew-1 isoform 1 or 2 is present. The discrimination between these two shrew-1 isoforms is not feasible with the currently available antibodies.

In contrast, the absence of antibody staining against the ED of shrew-1 in luminal cells suggests that only isoform 3 is present. In line with this, the murine mammary luminal epithelial cell line HC11 expresses only shrew-1 isoform 3. The main function of luminal cells is the differentiation into milk-secreting alveolar cells during pregnancy, a process driven by prolactin signaling via the JAK-STAT5 pathway (Cui et al., 2004; Gallego et al., 2001; Hennighausen et al., 1997). In parallel, localization of shrew-1 in luminal epithelial cells changes during lactation up to early involution. Here, the nuclear localization could suggest a participation in specific signaling and transcription events during differentiation and remodeling.

In contrast, the main functional feature of myoepithelial cells is to elicit structural support to the luminal cells by, for example, secreting basement membrane components, matrix metalloproteinases or paracrine signals and exerting contractile force mediated by oxytocin during lactation. They also mediate signals from the stroma to the luminal cells influencing their polarity (Adriance et al., 2005; Gudjonsson et al., 2005, 2002; Pandey et al., 2010). Shrew-1 isoform 1 and/or 2, the full-length transmembrane protein variants, show an overlapping distribution pattern with SMA, the contractile actin (ACTA2) in myoepithelial cells required for milk ejection (Haaksma et al., 2011). This could imply a role of shrew-1 isoform1 and/or 2 in mediation of signals to exert mechanical force or pressure for milk ejection.

In summary, we conclude from our data that shrew-1 exists in several protein variants depending on cellular and functional context. Although the transcripts for the three shrew-1 isoforms are detectable in human and murine organs, our analyses indicate cell type- and tissue-specific, as well as overlapping, expression patterns. These data are important observations for further analysis of shrew-1 gene products and their putative functional relevance. It is required to assess which shrew-1 isoforms are present within an analyzed cell type or tissue in order to unravel the individual subcellular targeting, function and/or regulation.

## MATERIALS AND METHODS

### Antibodies

Antibodies were purchased from BD Transduction Laboratories (Heidelberg, Germany; E-cadherin clone 36; IF 1:100, IB 1:1000), Sigma-Aldrich (Munich, Germany; AJAP1 HPA012157, IF 1:100, WB 1:1000; SMA clone 1A4, IF 1:100; pan-cadherin CH-19, IB 1:1000; myc C3956, IF 1:100, IB 1:1000), Ambion (Darmstadt, Germany; GAPDH clone 6C5, IB 1:10,000), Thermo scientific (Darmstadt, Germany; Grp94 clone 9G10.F8.2, IB 1:500), Abcam (Cambridge, UK; AJAP1 ab121361, IF 1:100, IB 1:1000), R&D Systems (Wiesbaden, Germany; AJAP1 AF7970), AntibodyVerify (Las Vegas, NV, USA; AJAP1 AAS47449C), Santa Cruz (Heidelberg, Germany; β-catenin H102, IB 1:1000; β-casein M-14, IB 1:1000) and custom made antibodies against shrew-1 (Nanotools, IB 1:500, polyclonal Genovac and monoclonal Genovac clone F, IF 1:50 and IB 1:500). Secondary antibodies (Alexa Fluor 488-, 594- and 647-labeled antibodies, IF 1:400) were purchased from Molecular Probes (Leiden, The Netherlands) or Abcam (Cambridge, UK) and Horseradish peroxidase (HRP)-conjugated secondary antibody from Jackson Immuno Research (Dianova, Hamburg, Germany) or Biosource (Camarillo, CA, USA).

### Mice, cell lines, culture and lactogenic differentiation

HEK293T cells (CRL-11268; ATCC, Manassas, VA, USA) and MCF-7 cells (European Collection of Cell Cultures, Salisbury, UK) were cultured in DMEM high glucose supplemented with 10% fetal calf serum (FCS), 100 U ml^−1^ penicillin and 100 µg ml^−1^ streptomycin (Sigma-Aldrich, Munich, Germany) at 37°C with 5% CO_2_. HC11 cells were cultured in RPMI supplemented with 10% heat-inactivated FCS, 5 µg ml^−1^ insulin, 10 ng ml^−1^ EGF and 100 U ml^−1^ penicillin and 100 µg ml^−1^ streptomycin (growth medium; Sigma-Aldrich, Munich, Germany) at 37°C with 5% CO_2_. For lactogenic differentiation, HC11 cells were grown to confluence and kept for further 2 days in growth medium. To induce differentiation, insulin was removed from the growing medium for 3 days before addition of the lactogenic hormone mix (DIP: 10^−7^ M dexamethasone, 5 µg ml^−1^ insulin, 5 µg ml^−1^ prolactin; Sigma-Aldrich, Munich, Germany) to the growing medium without EGF for 4 days as described (Taverna et al., 1991). All cell lines were *Mycoplasma-*negative. Female BALB/c mice (10 weeks of age) were purchased from Harlan Laboratories (Roßdorf, Germany) and all mouse procedures were performed in compliance with the German animal welfare law, guidelines and policies. Dissected Mammary gland tissue from Balb/c mice was minced and homogenized with ice-cold RIPA buffer (approx. 50 mg of sample in 300 µl ice cold RIPA buffer). From the cleared extract, 40 µg of total protein was separated by SDS–PAGE and immunoblotting.

### Plasmids and cloning

Shrew-1-myc constructs (isoform 1 and isoform 2) were generated by PCR using the vector pEGFP-shrew-1 (Bharti et al., 2004) as a template and cloned into pcDNA3.1(-) (Invitrogen, Karlsruhe, Germany) using *Nhe*I and *Acc65*I restriction sites. 5′-TTG GTA CCT TAC AGA TCC TCT TCT GAG ATG AGT TTT TGT TCG CAG GAG ATT TCA AAC CAT-3′ served as the reverse primer, tagging shrew-1 with a myc-tag at the C-terminus. The following oligonucleotides were used as forward primers: Iso1-shrew-1-myc: 5′-AAT TGC TAG CAT GTG GAT TCA ACA GCT T-3′, Iso2-shrew-1-myc: 5′- AAT TGC TAG CAT GTC CAT CCG CTG GC-3′. Shrew-1 isoform 3 was PCR-amplified from MCF-7 cells-derived cDNA (forward primer 5′- TTT AAG CTT ATG TGG ATT CAA CAG-3′; reverse primer 5′-TTG GTA CCT TAG CAG GAG ATT TCA AAC CAT TTC TC-3′) and cloned into pcDNA3.1(+) (Invitrogen, Karlsruhe, Germany) using *Hind*III and *Acc65*I restriction sites. To generate pEGFP-Golgi the sequence of the N-terminal signal-anchor of the human beta-1,4-galactosyl-transferase (GenBank Acc. No. AK293418.1) was PCR-amplified from HEK293T cells-derived cDNA (forward primer 5′-AAT TGC TAG CAT GAG GCT TCG GGA GCC GCT C-3′, reverse primer 5′-TTG GAT CCT TGG CCC CTC CGG TCC GGA GCT CCC C-3′) and fused N-terminally to the GFP in the pEGFP-N1 vector (Clontech, Saint-Germain-en-Laye, France) using the *Nhe*I and *BamH*I restriction sites. All constructs were checked by sequencing for integrity (SRD, Bad Homburg, Germany).

### Cell transfection

Cells were transfected with the transfection reagent jetPEI (PEQLAB Biotechnologie GmbH, Erlangen, Germany) as described (Resch et al., 2011). Briefly, 6×10^5^ HEK293T cells and 7×10^5^ MCF-7 cells, respectively, were seeded in 10 cm^2^ culture dishes. After one day, plasmid DNA was incubated with jetPEI and subjected to the cells according to the manufacturer's instructions. Cells were cultivated for the desired time.

### Cytosol-microsomal fractionation

Cytosol-microsomal fractionation was performed as described (Resch et al., 2008). Briefly, 24 h after transfection, cytosolic and microsomal fractions of cells (three cell culture dishes of 10 cm^2^) were isolated using the Qproteome Mitochondria Isolation Kit (Qiagen, Hilden, Germany). 20 µg of protein of each fraction was separated by SDS-PAGE and analysed by immunoblotting.

### Proteinase K protection assay

Microsomal fractions which were prepared on the very same day as described above were subjected to protein K protection assay. Therefore, 250 µl of the microsomal fractions (approx. 100 µg of protein) were supplemented with 2 µl of proteinase K solution (20 mg ml^−1^) (Fermentas, Darmstadt, Germany) and incubated on ice for 30 min. In a parallel reaction, 13 µl of 20% Triton X-100 solution was added. Both reactions were stopped by the addition of 5 µl 0.5 M EDTA solution (pH 7.6), 11 µl proteinase inhibitor cocktail Complete (Roche Diagnostics GmbH, Mannheim, Germany) and 93 µl pre-heated (95°C) protein sample buffer (Roti-Load 1; Carl Roth, Karlsruhe, Germany) followed by SDS-PAGE and immunoblotting.

### Biotinylation and precipitation of cell surface proteins

Cell surface biotinylation experiments were performed as described (Resch et al., 2011). Briefly, 24 h after transfection, cells were washed with ice cold PBS pH 8.0 and incubated with 0.5 mg ml^−1^ EZ-Link Sulfo-NHS-LC-Biotin (Thermo Scientific, Darmstadt, Germany) diluted in PBS pH 8.0 on ice for 1 h. Cells were washed twice with ice cold PBS pH 8.0 and once with ice cold 25 mM Tris-HCl, pH 8.0, lysed with 200 µl RIPA buffer (150 mM NaCl, 50 mM Tris-HCl, pH 7.5, 0.5% sodium deoxycholate, 1% Nonidet P-40, 0.1% SDS) containing proteinase inhibitor cocktail Complete (Roche Diagnostics GmbH, Mannheim, Germany). Cleared cell lysates with a total protein amount of 150 µg adjusted to a concentration of 1 µg µl^−1^ were incubated under rotation with 100 µl of a 50:50 slurry of NeutrAvidin agarose resins (Thermo Scientific, Darmstadt, Germany; resuspended in RIPA buffer) overnight. The supernatants were collected and the resins were washed three times with RIPA buffer, supplemented with protein sample buffer (Roti-Load 1, Carl Roth, Karlsruhe, Germany) and subjected to SDS-PAGE and immunoblotting.

### Immunoblotting

In general, separated proteins were transferred onto nitrocellulose or PVDF membranes in a semi-dry blotting chamber (PEQLAB Biotechnologie GmbH, Erlangen, Germany). A pre-stained protein standard was used as a molecular size marker (Fermentas, Darmstadt, Germany). Membranes were blocked with 5% nonfat milk powder or 5% BSA in Tris-buffered saline Tween-20 (TBST: 10 mM Tris–HCl, pH 7.4, 150 mM NaCl and 0.05%Tween-20) for 1 h. After a single wash step with TBST, the membranes were incubated with primary antibody overnight at 4°C, and after intensive washing, the bound primary antibody was detected with HRP-conjugated secondary antibodies (Dianova, Hamburg, Germany) and HRP substrate solution (2.5 mM luminol, 0.4 mM p-coumaric acid, 100 mM Tris–HCl, pH 8.5, and 0.009% H_2_O_2_).

### Immunocytochemistry and immunohistochemistry

Cells were seeded at a density of 100,000 cells per well onto coverslips (18 mm diameter). Coverslips were washed once with pre-warmed PBS, fixed for 10 min in 4% PFA, permeabilized for 10 min (0.5% Triton X-100 in PBS) and blocked for 30 min in 10% FCS in PBS (Sigma-Aldrich, Munich, Germany). Specific antigens were detected by incubation with primary antibodies overnight at 4°C in 10% FCS in PBS followed by incubation with 10 μg ml^−1^ of Alexa Fluor-labeled secondary antibodies from donkey for 45 min at room temperature in the dark, counterstained for 5 min with 1.5 μg ml^−1^ DAPI in PBS and mounted in Mowiol (Carl Roth, Karlsruhe, Germany). Immunohistochemical analysis was performed on formalin-fixed paraffin sections (5 μm), cleared in xylene, rehydrated through an alcohol series followed by Antigen retrieval (10 mM sodium citrate buffer pH 6) using a pressure cooker (3 min, setting 2). Sections were blocked in 10% FCS in TBS (Sigma-Aldrich, Munich, Germany) for 1 h. Primary antibodies were incubated in 10% FCS/TBS overnight at 4°C followed by incubation with 10 μg ml^−1^ Alexa Fluor-conjugated secondary antibody from donkey (Invitrogen GmbH, Karlsruhe, Germany) for 2 h at room temperature in the dark. Sections were counterstained with 1.5 μg ml^−1^ DAPI in PBS for 10 min and mounted in Mowiol. Staining was assessed by using a Zeiss LSM780 confocal microscope and Zen software. Images were processed by Fiji software (Schindelin et al., 2012).

### RNA isolation, cDNA synthesis and shrew-1 transcript variant analysis

RNA isolation was performed using the RNA Extract II kit (Macherey & Nagel, Düren, Germany), according to the manufacturer's instructions. RNA was eluted with RNase-free ddH_2_O and stored at −80°C. cDNA synthesis was carried out using the M-MLV reverse transcriptase (Promega, Mannheim, Germany) according to the manufacturer's instructions. Shrew-1 transcript variants were detected by PCR in human cDNA libraries of different organs and tissues (Clontech, Saint-Germain-en-Laye, France). For the detection of human shrew-1 Exon1-specific oligonucleotide (F_exon1_27mer) 5′-TCT GAG GCC CCG CTC CCC GAA ACG TGA-3′ and Exon1a-specific oligonucleotide (F_exon1a_29mer) 5′-ATC CGG AGC AGA TCT CAT TTC CCT GAG TA-3′ were used as forward primers, Exon5-specific oligonucleotide (R_3UTR_28mer) 5′-GGC GTC TGC CCT GCC CCC AGG AGG TAA A-3′) was used as the reverse primer. GAPDH was detected as a positive control with following primers: F_GAPDH 5′- TGA AGG TCG GAG TCA ACG GAT TTG GT-3′, R_GAPDH 5′-CAT GTG GGC CAT GAG GTC CAC CAC-3′. For the detection of murine shrew-1 Exon1-specific oligonucleotide (F_exon1_mur) 5′-GTG ACC ATG TGG ATC CAA CAG C-3′ and Exon4-specific oligonucleotide (F_exon4_mur) 5′-ACA CTC GGA GGA ATA GCC ACC A-3′ were used as forward primers, Exon5-specific oligonucleotide (R_3UTR_mur) 5′-AGG AGG TAA AAA GCC TTC GGC-3′) was used as the reverse primer. BIP was detected as a positive control with following primers: F_BIP 5′- TAC ACT TGG TAT TGA AAC TG-3′, R_BIP 5′-GGT GGC TTT CCA GCC ATT C-3′.

### Neuraminidase and O-glycanase protein digestion

To remove O-linked glycans from proteins, whole cell lysates in RIPA buffer were treated with Neuraminidase (New England Biolabs GmbH, Frankfurt am Main, Germany; Cat. No. P0720S) and Endo-α-N-acetylgalactosaminidase, also known as O-glycanase (New England Biolabs; Cat. No. P0733S), according to the manufacturer's instructions. Therefore, a total protein amount of 90 μg was adjusted with RIPA buffer to a final volume of 45 μl, followed by the addition of 5 μl of 10× Glycoprotein Denaturing Buffer (5% SDS, 0.4 M DTT). Proteins were denatured by heating at 95°C for 10 min. The chilled reaction was supplemented with 7 μl G7 Buffer (0.5 M sodium phosphate, pH 7.5) and 7 μl of 10% NP-40. The reaction was divided in to three equal parts. One third was supplemented with 2 μl ddH2O giving the negative control. The second third was supplemented with 1 μl ddH2O and 1 μl Neuraminidase (50 U/μl). The last third was supplemented with 1 μl Neuraminidase and 1 μl O-glycanase (40,000 U/μl-1). All reactions were incubated at 37°C for 3.5 h. The reactions were stopped by addition of protein sample buffer (Roti-Load 1; Carl Roth, Karlsruhe, Germany, K929.1).

### IHCP with antibody preabsorption

Preabsorption of the antibody against the cytoplasmic domain of shrew-1 (ab121361, abcam) was performed according to the manufacturer's instruction (PREST protocol, Atlas Antibodies, Stockholm, Sweden). Briefly, the primary antibody mixture (shrew-1 ab121361 and anti-SMA) together with shrew-1 peptides (5 μl each of *in vitro* translated human shrew-1 protein aa1-411 and aa1-282 together with 30 μl GST-Shrew-1 aa 304-411) was incubated rotating overnight at 4°C followed by a centrifugation step [(15 min at 16.8 ***g*** (13200 rpm)] to pellet immune complexes prior proceeding with the immunohistology protocol. As a control, the primary antibody mixture without the addition of shrew-1 peptides was treated in the same way. The addition of the anti-SMA antibody served as an internal control for successful staining procedure. Staining was assessed using a Zeiss LSM780 confocal microscope and Zen software (http://www.zeiss.de/microscopy/de_de/produkte/mikroskopsoftware/zen.html#downloads). Images were processed by Fiji software (Schindelin et al., 2012).

### Computational analysis

The presence and location of signal peptide cleavage sites were analyzed with SignalP (http://www.cbs.dtu.dk/services/SignalP/; Petersen et al., 2011), putative splice sites were analyzed with Splign (http://www.ncbi.nlm.nih.gov/sutils/splign/splign.cgi; Kapustin et al., 2008) and O-glycosylation sites were identified with NetOGlyc (http://www.cbs.dtu.dk/services/NetOGlyc/; (Julenius et al., 2005).
